# Patient profile and antibiotic use in a dedicated upper respiratory tract infection clinic based in a primary healthcare setting during COVID-19 pandemic in Malaysia: A cross sectional study

**DOI:** 10.51866/oa.38

**Published:** 2022-08-25

**Authors:** Zhi Yin Ooi, Nurul Abidah Mohd Ghazali, Nang Juniza Nik Zahari, Huan Keat Chan, Norsiah Md Noor, Noor Liani Harun, Mohd Firdaus Abu Bakar, Mohd Redhuan Abdul Muin

**Affiliations:** 1 MD (UNIMAS), Klinik Kesihatan Taman Universiti, Jalan Kebudayaan 28, Taman Universiti, Johor Bahru, Johor, Malaysia. Email: ooizhiyin0704@gmail.com; 2 MD (UMS), Hospital Sultanah Bahiyah, Km 6, Jln Langgar, Bandar, Alor Setar, Kedah, Malaysia.; 3 MB BCh BAO (IRELAND), Doc of Fam Med (UKM), Klinik Kesihatan Bandar Alor Setar, Aras 1 Blok A, Darul Aman Highway, Alor Setar, Kedah, Malaysia.; 4 MSc (USM), Clinical Research Centre, Hospital Sultanah Bahiyah, Km 6, Jln Langgar, Bandar, Alor Setar, Kedah, Malaysia.; 5 MBBS (MAL), M.MED (FAMILY MEDICINE) UKM, Klinik Kesihatan Bandar Alor Setar, Aras 1 Blok A, Darul Aman Highway, Alor Setar, Kedah, Malaysia.; 6 MBBS (UM), Doc of Fam Med (UKM), Klinik Kesihatan Bandar Alor Setar, Aras 1 Blok A, Darul Aman Highway, Alor Setar, Kedah, Malaysia.; 7 MBBS (IIUM), Klinik Kesihatan Bandar Alor Setar, Aras 1 Blok A, Darul Aman Highway, Alor Setar, Kedah, Malaysia.; 8 MB BCh BAO (IRELAND), Klinik Kesihatan Bandar Alor Setar, Aras 1 Blok A, Darul Aman Highway, Alor Setar, Kedah, Malaysia.

**Keywords:** Patient profile, URTI clinic, COVID-19, Primary care, Antibiotic

## Abstract

**Introduction:**

Upper respiratory tract infection (URTI) is commonly encountered at the primary care level. Its management is particularly challenging due to the similarity of its symptoms to coronavirus disease 2019 (COVID-19) infection. Our study evaluated the profiles and antibiotic use of patients seeking care from a dedicated community-based URTI clinic during the COVID-19 pandemic.

**Method:**

A cross-sectional study was conducted. Data were obtained from the medical records of patients visiting the URTI clinic at the Alor Setar Primary Healthcare Centre between March and April 2020.

**Results:**

Overall, 587/4388 (13.3%) patients received treatment at the URTI clinic. Most patients were male (60.6%) and aged between 20 and 39 years (35.5%). Their most common symptoms were cough (68.4%), fever (31.6%), runny nose (24.6%), and sore throat (24.1%). Most patients were diagnosed with acute nasopharyngitis (52.5%), acute pharyngitis (18.6%), or acute tonsillitis (5.3%). The symptomatic medication prescription rate was 96.5%. Only 26 of the 435 patients diagnosed with URTI received antibiotics, yielding an antibiotic use rate of only 6.0% for URTI relative to overall drug use. Acute tonsillitis was more common in children <12 years old (p<0.001), while a cough and runny nose were more commonly indicative of acute nasopharyngitis than other conditions (p<0.001). Sore throat was more likely to be a symptom of acute pharyngitis (p<0.001) and acute tonsillitis (p<0.001).

**Conclusion:**

Despite the challenges faced during the COVID-19 pandemic, the findings suggest that patients with URTI-like symptoms were properly managed, and the rate of antibiotic usage remained reasonable.

## Introduction

Upper respiratory tract infection (URTI) is characterised by an infectious process involving the upper respiratory tract, including the nose, para-nasal sinuses, pharynx, larynx, or trachea.^1^ The National Medical Care Statistics (NMCS) 2014 by the Ministry of Health Malaysia and several other local studies reported that respiratory conditions were the most common medical problem seen in primary care, ranging from 26.8% to 37.2% of all problems.^2-4^ In Singapore, surveys of primary healthcare clinics conducted in 2010 and 2014 found that URTIs were the dominant disease diagnosed in primary healthcare centres, comprising 25% of all diagnoses made in 2010 and 20% in 2014.^5^ In 2013, 18.8 billion cases of URTI were recorded worldwide.^6^

Currently, the world is stuck by a novel viral pandemic known as coronavirus disease 2019 (COVID-19). The pandemic not only threatens global health but impacts nearly every country, including Malaysia. The World Health Organisation (WHO) declared the outbreak of novel coronavirus as a pandemic on 11 March 2020.^7^ Diagnosing URTI can be challenging during the COVID-19 pandemic. COVID-19 can have wide spectrum of symptoms, including fever (76.5–98.6%), cough (50.0–70.0 %), flu-like symptoms (4.0–4.8%), and sore throat (1.7–4.0%), all overlapping with URTI symptoms.^8,9^ Our clinic implemented a new URTI clinic as one of the methods of preventing COVID-19 transmission in line with the Centres for Disease Control and Prevention (CDC) guideline entitled ‘Interim Infection Prevention and Control Recommendations for Patients with Suspected or Confirmed Coronavirus Disease 2019 in Healthcare Settings’; one of the key concepts is to limit how germs can enter the facility.^10^

All patients who attend our facility are screened for suspected COVID-19 at triage and are stratified into low or high risk for COVID-19 in accordance with the 2020 Ministry of Health Malaysia guidelines on COVID-19 management.^11^ Low-risk COVID-19 patients are seen in the URTI clinic, while highly suspected patients are diverted to the COVID-19 swab team. This is crucial for several reasons: first, a suspected case of COVID-19 needs to be isolated promptly to halt further spread of the virus;^11^ next, the usage of personal protective equipment needs to be optimised to prevent waste.^9^ These considerations highlight how challenging a balanced approach is to a patient with a URTI during COVID-19 pandemic.

This study will help to outline patterns in the sociodemographic and clinical characteristics of patients attending the URTI clinic in addition to practices among healthcare providers regarding diagnosis and antibiotic usage for treating URTIs in patients attending Alor Setar Primary Healthcare Centre during the COVID-19 pandemic. Our study will also investigate the association between sociodemographic characteristics and diagnosis in addition to the common presentations and diagnosis of URTI. These results will aid primary care physicians in identifying opportunities for intervention, such as minimising unnecessary prescription of antibiotics and symptomatic drugs, and proper allocation of resources for other medical illness during pandemic.

## Methods

A cross-sectional study was conducted at Alor Setar Health Clinic (also known as Klinik Kesihatan Bandar Alor Setar, or KKBAS), a large government polyclinic in the district of Kota Setar in Kedah that provides subsidised general outpatient, maternal, and child health services for the residents of Kota Setar, Kedah—a population of 366 787 according to figures from the 2010 census.^12^ The total patient attendance at KKBAS from 23 March 2020 to 28 April 2020 was 4 388; of these patients, 587 (13.3%) were seen in the URTI clinic. All patients attending KKBAS were stratified to low or high risk for COVID-19. High risk for COVID-19 was suspected when a patient presented to the triage counter with the following features: attended an event or visited an area associated with known COVID-19 cluster or red zone OR travelled to or resided in a foreign country within 14 days of the onset of illness OR close contact 14 days before illness onset with a confirmed case of COVID-19. Low-risk COVID-19 patients with respiratory symptoms were seen in the URTI clinic.

All eligible patients were included in the study, and sampling was not conducted. The cases involved in this study were identified by reviewing the medical records of patients who attended the URTI clinic between 23 March 2020 and 28 April 2020. All the patients who attended the URTI clinic were marked, and only those fulfilling the criteria were selected and analysed. Patients who attended the clinic for medication prescription refills, patients with high risk of COVID-19 infection, and patients with medical records with incomplete information were excluded from this study.

The medical records from the study period were retrieved from the medical record room. The information extracted and recorded from these medical records included basic demographic details, clinical symptoms and signs, physical examination results, investigations, diagnoses, and management provided. The collected data was then analysed using SPSS software version 12, and appropriate statistical calculations for qualitative and quantitative data were used to determine whether the results were significant, indicated by a p value less than or equal to 0.05. Descriptive statistics were employed for selected variables. The findings are presented based on the type of data and their distribution: categorical data are presented as frequencies and percentages, and numerical data are presented as means and standard deviations if normally distributed and as medians and interquartile ranges if not normally distributed.

Pearson’s chi-square test was used to compare the distribution of a categorical variable in a sample or a group with its distribution in another group. If the distribution of the categorical variable was not different between different groups, we concluded that the distribution of the categorical variable was not related to the variable of groups. Fisher’s exact test was used to detect non-random associations between two categorical variables. Pearson’s chi-square test and Fisher’s exact test were used to determine the association between diagnosis and sociodemographic characteristics in addition to the association between common presentation and diagnosis. The significance level was set at P < 0.05.

## Results

### 3.1 Sociodemographic characteristics

Over a 1-month period, 4 388 patients visited the outpatient clinic of KKBAS from 23 March 2020 to 28 April 2020; 587 (13.3%) of the patients sought treatment in the URTI clinic. A total of 564 patients met the inclusion criteria, while 23 patients were excluded, giving an overall proportion of patients attending the URTI clinic of 564/4 388 (13.3%). Most of the patients who attended the URTI clinic were male (342/564, 60.6%), and the most common age group was 20–39 years old (222/564, 35.5%). The mean age of the patients who attended the URTI clinic was 41.2±21.9 years. A total of 84 cases (15.0%) were children (aged ≤18 years old) and 480 cases (85.0%) were adults; 67 (11.9%) of the patients were aged <9 years, while 139/564 (24.6%) were elderly, aged >60 years old, which was in line with the recommendations of the Ministry of Health Malaysia.

Common comorbidities, such as diabetes mellitus, hypertension, ischaemic heart disease, bronchial asthma, or chronic obstructive pulmonary disease (COPD) were included in this study. The most common positive findings in the medical history of the patients who visited the URTI clinic were hypertension (108/564, 19.1%) and diabetes mellitus (72/564, 12.8%). The demographic details of the study population are summarised in **Table 1**.

**Table 1:** Demographic and clinical characteristics of the study population (n=564).

Characteristic	n	%
*Age, years* ^a^		
0-9	67	11.9
10-19	19^b^	3.4
20-29	88	15.6
30-39	112	19.9
40-49	54	9.6
50-59	85	15.1
60 69	80	14.2
70-79	46	8.2
80-89	13	2.3
*Gender*		
Male	342	60.6
Female	222	39.4
*History of illness^c^*		
Diabetes mellitus	72	72/564, 12.8%
Hypertension	108	108/564, 19.1%
Ischaemic heart disease	14	14/564, 2.5%
Asthma or COPD	36	36/564, 6.4%

COPD, chronic obstructive pulmonary disease.

a Mean age = 41.2±21.9 years.

b Out of 19 patients in the age category 10–19 years, 2 were 19 years old.

c Patients without comorbidities are not included in the table. A patient could have more than one illness.

### 3.2 Common symptoms, diagnosis of patients, and antibiotic prescription rate in the URTI clinic

Most of the patients presented with various symptoms and reported having multiple symptoms during their visit to the URTI clinic (**Table 2**). The most common symptoms were cough (68.4%), fever (31.6%), runny nose (24.6%), and sore throat (24.1%). Only one patient reported anosmia (0.2%) and was treated as acute nasopharyngitis. Chest pain (2.0%) and shortness of breath (5.3%) were rare. ICD-10 codes for acute respiratory infection were used to record the provisional diagnosis after consultation on the clerking sheet. The diagnoses included 1- acute nasopharyngitis, 2- acute sinusitis, 3- acute pharyngitis, 4- acute tonsillitis, 5- acute laryngitis and tracheitis, 6- acute obstructive laryngitis and epiglottitis, 7- acute bronchitis, 8- acute bronchiolitis, 9- asthma, 10- COPD, 11- bronchiectasis, 12- viral pneumonia, 13- bacterial pneumonia, and 14- others. The most common diagnosis for the patients seen in the URTI clinic were acute nasopharyngitis (52.5%), acute pharyngitis (18.6%), and acute tonsillitis (5.3%). The other patients were diagnosed with acute bronchitis (0.7%), acute sinusitis (0.7%), asthma (2.1%), or other (20.0%). URTI was diagnosed in 435 patients (435/564, 77.1%).

**Table 2:** Symptoms of patients (n=564) and diagnoses in the URTI clinic of KKBAS.

Characteristic	n^a^	%
*Symptoms*		
Cough	385	68.3
Runny nose	139	24.6
Sore throat	136	24.1
Anosmia	1	0.2
Fever	178	31.6
Chest pain	11	2.0
Shortness of breath	30	5.3
*Diagnosis*		
Acute nasopharyngitis	296	52.5
Acute pharyngitis	105	18.6
Acute tonsillitis	30	5.3
Acute bronchitis	4	0.7
Acute sinusitis	4	0.7
Asthma	12	2.1
Other	113	20.0

a One patient could have more than one symptom.

A total of 544 patients (544/564, 96.5%) were provided with symptomatic treatment in the URTI clinic, which included antipyretics (51.8%), antihistamines (49.8%), and anti tussive agents (45.9%) (**Table 3**). The mean number of medications prescribed per visit was 1.6. Of the 564 patients who visited the URTI clinic, only 35 (6.2%) were prescribed an antibiotic; 26 patients (26/435, 6.0%) diagnosed with URTI were prescribed an antibiotic, making the prescription rate of antibiotics among URTI patients acceptably low. Among all antibiotics, the penicillin group was the most prescribed, constituting of 68.57% (n=24) of the total antibiotics prescribed for all cases, followed by macrolides (erythromycin ethyl succinate). Augmentin was the least prescribed antibiotic, representing 5.7% of prescriptions for URTI.

**Table 3:** Type of treatment provided to the patient (n=564) in the URTI clinic of KKBAS.

Type of Treatment Provided	n^a^	%	Current evidence
Antipyretic	292	292/564, 51.8	May help in fever
Antitussive	259	259/564, 45.9	May reduce symptoms
Antihistamine	281	281/564, 49.8	Not effective
Antibiotic^b^	35^c^	35/564, 6.2	Little evidence of benefit in uncomplicated URTI

a One patient could have received more than one type of treatment

b The type of antibiotics used: amoxicillin (n=24), erythromycin ethyl succinate (n=6), amoxicillin/clavulanate (n=2), and others (n=3).

c 26 out of 35 patients prescribed with an antibiotic were diagnosed with URTI.

### 3.3 Bivariate analysis of association

Pearson’s chi-square test revealed a significant association between acute tonsillitis and children <12 years old (p<0.001). However, no significant association was reported among other study variables (**Table 4**).

**Table 4:** Characteristics associated with the diagnoses of the patients.

Characteristic	Acute nasopharyngitis		Acute pharyngitis		Acute tonsillitis		Others	
	n (%)	p^a^	n (%)	p^a^	n (%)	p^a^
*Age, years*								
<12	31 (43.1)	0.230	14(19.4)	0.422	11 (15.3)	<0.001	16(22.2)	0.875
12-64	214(53.9)		69(17.4)		18(4.5)		96 (24.2)	
≥65	51 (53.7)		22 (23.2)		1 (1.1)		21 (22.1)	
*Gender*								
Male	181 (52.9)	0.794	60(17.5)	0.416	18 (5.3)	0.941	83 (24.3)	0.633
Female	115(51.8)		45 (20.3)		12(5.4)		50 (22.5)	
*Diabetes mellitus*								
No	254 (51.6)	0.287	94 (19.1)	0.436	28 (5.7)	0.408^b^	116(23.6)	0.995
Yes	42 (58.3)		11 (15.3)		2 (2.8)		17(23.6)	
*Hypertension*								
No	237 (52.0)	0.619	84 (18.4)	0.806	26 (5.7)	0.405	109 (23.9)	0.711
Yes	59 (54.6)		21 (19.4)		4 (3.7)		24 (22.2)	
*Ischaemic heart disease*								
No	288 (52.4)	0.724	102(18.5)	0.732^b^	30 (5.5)	>0.95^b^	130 (23.6)	>0.95^b^
Yes	8(57.1)		3(21.4)		0 (0.0)		3(21.4)	
*Asthma/chronic obstructive pulmonary disease*								
No	282 (53.4)	0.091	100(18.9)	0.451	29 (5.5)	0.7 \2^l^	117(22.2)	0.002
Yes	14 (38.9)		5(13.9)		1 (2.8)		16(44.4)	

a Pearson’s chi-square test.

b Fisher’s exact test.

The symptoms of cough and runny nose were significantly associated with the diagnosis of acute nasopharyngitis (p <0.001) and non-URTI-related disease (p<0.001). The patients with sore throat were significantly more likely to receive a diagnosis of acute pharyngitis, acute tonsillitis, or other (p<0.001). Fever was significantly associated with diagnosis of acute tonsillitis (p=0.001), and shortness of breath was significantly associated with acute nasopharyngitis (p=0.001).

**Table 5:** Symptoms associated with the diagnoses of the patients.

Symptom	Acute nasopharyngitis		Acute pharyngitis		Acute tonsillitis		Others	
	n (%)	p^a^	n (%)	p^a^	n (%)	p^a^
*Cough*								
No	65 (36.3)	**<0.001**	28(15.6)	0.216	12 (6.7)	0.318	74(41.3)	**<0.001**
Yes	231 (60.0)		77 (20.0)		18(4.7)		59(15.3)	
*Runny nose*								
No	**206 (48.5)**	0.001	82 (19.3)	0.470	22 (5.2)	0.792	115 (27.1)	**0.001**
Yes	90 (64.7)		23 (16.5)		8 (5.8)		18(12.9)	
*Sore throne*								
No	226 (52.8)	0.786	62(14.5)	**<0.001**	13 (3.0)	**<0.001**	127 (29.7)	**<0.001**
Yes	70 (51.5)		43(31.6)		17(12.5)		6 (4.4)	
*Anosmia*								
No	295 (52.4)	>0.95^b^	105 (18.7)	>0.95^b^	30 (5.3)	>0.95^b^	133 (23.6)	>0.95^b^
Yes	1 (100.0)		0 (0.0)		0 (0.0)		0 (0.0)	
*Fever*								
No	206 (53.4)	0.535	71 (18.4)	0.841	12 (3.1)	**0.001**	97 (25.1)	0.202
Yes	90 (50.6)		34(19.1)		18(10.1)		36 (20.2)	
*Chest pain*								
No	292 (52.8)	0.280	104 (18.8)	0.699^b^	30 (5.4)	>0.95^b^	127 (23.0)	**0.025** ^b^
Yes	4 (36.4)		1 (9.1)		0 (0.0)		6 (54.5)	
*Shortness of breath*								
No	289 (54.1)	**0.001**	101 (18.9)	0.445	30 (5.6)	0.395^b^	114 (21.3)	<0.001
Yes	7 (23.3)		A (13.3)		0 (0.0)		19(63.3)	

a Pearson’s chi-square test.

b Fisher’s exact test.

## Discussion

There was a relatively lower attendance (13.3%) of patients with respiratory symptoms during the COVID-19 pandemic in our study compared with other studies, as patients with influenza-like illness and suspected cases of COVID-19 were advised to visit designated COVID-19 swab areas. The gender distribution in our study is proportionate to the estimation population of Kedah in 2019, which was 2.18 million in total: 1.11 million males (50.9%) and 1.07 million females (49.1%).^12^ However, this observation is inconsistent with the findings of the NMCS 2014, which reported that more females than males used services at public primary care facilities in Malaysia.^4^ The beginning of the Movement Control Order (MCO) period might lead to the reduction in visits to congested places, such as government clinics, because females were more incline to view themselves at risk for COVID-19 than males.^13^

The younger, 20–39-year-old group of patients frequently seeks treatment in primary care for mild respiratory symptoms. This age group engages in more social activities in person and contracts upper respiratory tract infection easily.^14^ In addition, the younger generation is exposed to social media, which has become a first-hand information channel during the pandemic, and they have greater awareness of the disease during this period.^15,16^ Interestingly, 76% of confirmed COVID-19 cases were diagnosed in adults less than 65 years of age, with most aged 18-29 years;^17,18^ this observation is consistent with our study results. Furthermore, the paediatric group aged <9 years (11.6%) accounted for a small proportion in our clinic, in contrast with the data reported by the Upper Respiratory Tract Infection Expert Meeting Consensus, which claimed that over 50.0% of visits to a primary care centre were paediatric visits according to a survey of primary medical clinics in Singapore.^5^ This can be explained by the implementation of the MCO, whereby the government implemented national school closures related to COVID-19, which resulted in a potentially significant reduction in transmission of URTIs among school-aged children. In contrast, adults with comorbidities comprised most of the attendees at the URTI clinic as they were prone to respiratory tract infections or COVID-19 due to their immune-compromised state.^19^

The diagnosis of acute respiratory infection in this study was based on the ICD-10 codes, which was different from several previous studies in Malaysia in which the infections were classified based on the International Classification of Primary Care.^3,20,21^ Compared to a study by Hak et al. (2006), the five most common URTI diagnoses presenting to the general practitioner in the Netherlands were acute rhinitis, acute sinusitis, acute bronchitis, acute otitis media, and acute tonsillitis.^21^ This inconsistency might be due to different coding methods and unfamiliarity of the attending medical officers with the disease classification system. Classification is difficult in primary care, especially for vague and symptom-based conditions. Training should be provided to all primary care physicians to enhance the quality of data collected based on the ICD-10.

The occurrence of acute tonsillitis is more common in preteen children (<12 years old). Similar observations from studies by Hidaya Qarqani Bukhari et al. (2019) and Middleton et al. (1988) reported that most tonsillitis cases from the 6-12-year-old age group were due to low and immature immunity in this age group.^22,23^ Cough, fever, runny nose, and sore throat remained the most common symptoms experienced by patients visiting the URTI clinic. All the symptoms were significantly associated with a URTI diagnosis. Moreover, most patients with a sore throat had pharyngitis and acute tonsillitis.^24,25^ This suggests the utility and accuracy of ICD-10 coding for URTIs. Specific infectious agents resulting in a respiratory disease are difficult to identify at the initial visit. Therefore, the presence of classical features or symptoms that are statistically associated with URTIs in the absence of warning signs is sufficient to guide the diagnosis. Diagnostic testing, which is not readily available in our setting, is usually not necessary.^1^

There was no conclusive evidence to suggest anosmia as a clinical feature of COVID-19 during the early outbreak of pandemic, with one Chinese study reporting that 5.1% of affected patients had anosmia.^8^ Subsequently, several later literature reviews concluded that a significant number of COVID-19 patients (53%) had symptoms of anosmia.^26^ As COVID-19 resources are limited, universal testing for anyone with signs and symptoms of COVID-19, as recommended by the CDC (2021), was not widely implemented in the early COVID-19 era.^10,24^ Our country still relies on epidemiological features, including contact with a confirmed COVID-19 case, to suspect the disease. Therefore, patients with anosmia were treated as URTIs in our clinic. Viral URTI with runny nose and nasal congestion may contribute to conductive olfactory loss.^27^ During the early phase of COVID-19, this overlapping symptomatology was a diagnostic challenge and further hindered accurate diagnosis and early containment.

Shortness of breath is usually a cardinal sign suggesting more sinister pathologies; however, it can also be seen in acute upper respiratory infections.^28^ Interestingly, our study found that shortness of breath was statistically associated with acute nasopharyngitis. Shortness of breath, a subjective syndrome of breathing discomfort that varies in intensity, is associated with interactions between multiple factors, included physiological, psychological, social, and environmental factors.^29^ With an aggravation of breathing discomfort in URTI, patients may consider breathlessness as a threat associated with anxiety or depressive symptoms, which may induce secondary physical responses. A study regarding public psychological and behavioural responses during the COVID-19 outbreak reported that a greater proportion of the public may have experienced anxiety disorders as the COVID-19 outbreak progressed.^30^ The public is more likely to express higher anxiety levels if they perceive they are at risk of becoming infected with COVID-19.^31^

It is important to understand the pathophysiology of the symptoms of URTI, as most treatments for URTI focus on symptomatic relief as the symptoms are perceived as a nuisance despite the self-limiting nature of the illness; antibiotic usage is thus unnecessary. A study found that 96.6% of cases were prescribed symptomatic drugs, similar to the 98% URTI drug prescription patterns of Hong Kong doctors.^32^ Possible explanations for a higher symptomatic prescription rate may be related to the nature of our healthcare system, where consultation fees are heavily subsidised and include the cost of medication, patients’ relatively low self-medication rates, and high expectations for receiving a prescription for medication during consultations despite the self-limiting nature of the illness. However, there is limited evidence that these medications are effective for URTI, as shown in **Table 3**.^32^

The URTI-specific antibiotic prescription rates in the Netherlands and Hong Kong in 2010 were 17% and 5%, respectively.^32,33^ From National Medical Care Study (NMCS) 2010 data, 46.2% of patients diagnosed with URTI reported receiving antibiotic treatment. This data includes 16.8% of public and 57.7% of private clinic patients.^20^ The antibiotic prescription rate for URTI in our study was much lower than previous studies. Guidance based on the local National Antimicrobial Guidelines from 2019^34^ includes a modified Centor Score playing a role in judicious antibiotic use among public primary care doctors. A standard approach for assessment and management of patients presenting with respiratory tract infection and the role of antibiotics based on best available evidence is needed to avoid overuse of antibiotics for URTIs in the COVID-19 era.^35^ Choices of antibiotics in this study were consistent with the NMCS 2010; the most prescribed antibiotics for URTI were amoxicillin, cephalexin, and erythromycin, at rates of 35.0%, 15.1%, and 12.0%, respectively.^36^

Overall, compared to the non-pandemic studies on URTIs, the characteristics of patients have changed. For example, the paediatric group <12 years old were the dominant group (>1/3) pre-pandemic, while in our study, the 20–39-year-old age group visited the URTI clinic most often (>1/3).^2^ This implies that there might be changes in healthcare-seeking behaviour by symptom and age during the pandemic. Antibiotic usage remained low, and symptomatic medication prescription rates were high both pre-pandemic and during the pandemic. Analysing the characteristics of patients who utilised primary care during the pandemic, targeted patient education should be provided via public health promotion to empower selfmanagement and reasonable indication for medical attention during the pandemic. As there is limited evidence for the benefit of symptomatic treatment in URTI, with heavy cost to public health, this issue should also be addressed. Gaps have been highlighted between primary care practice and evidence-based management of URTIs. This study was unable to ascertain COVID-19 among URTI patients. Key clinical features that may guide in differentiating a COVID-19 case, which require specific testing, from upper respiratory and/or influenza-like illnesses of other aetiologies were not determined in this study.

## Conclusion

Although the number of COVID-19 cases is still increasing in Malaysia and the pandemic has yet to reach its peak, URTI patients are properly managed during the pandemic. Low antibiotic prescription rate indicate judicious antibiotic use among public primary care doctors, particularly in the study region, during the pandemic era. The high number of symptomatic drug prescriptions and the relationship with patients’ expectation and doctors’ prescription habits during consultations should be further explored. From the study of association between sociodemographic characteristics and diagnosis, URTI remains common in the younger age group (<12 years old). Diagnosis and common symptoms of URTI are correlated with high significance. Our URTI clinic is another line of defence that was implemented to improve detection and prevent further spread of COVID-19 by understanding patient profiles to aid in best management practices, improve healthcare resource utilisation, adequately protect the community, and provide optimal care to patients with URTIs during the COVID-19 pandemic. Furthermore, this study highlights the need for symptom-based screening or better application of diagnostic tests during the pandemic.
